# Effect of vasoactive drugs on the response of the baroreceptor and regulation of heart rate variability in patients with septic shock

**DOI:** 10.1186/cc12662

**Published:** 2013-06-19

**Authors:** AC Nogueira, V Kawabata, P Lotufo, L Ferandes, R Brandão, R Jenner, C Mostarda, MC Yrigoyen, H Barbeiro, FG Soriano

**Affiliations:** 1 Hospital Universitário da Usp, Butantã, São Paulo, SP, Brazil

## Introduction

We evaluated the action of vasoactive drugs on baroreceptor regulation and the interaction of this in regulation of heart rate variability (HRV) in patients with septic shock.

## Methods

A prospective observational study of patients with severe sepsis or septic shock. Collected data were analyzed retrospectively, separating patients according to their clinical evolution: survival or death. We monitored troponin I, vasoactive drug dose, HRV and blood pressure variability.

## Results

**Figure 1:**
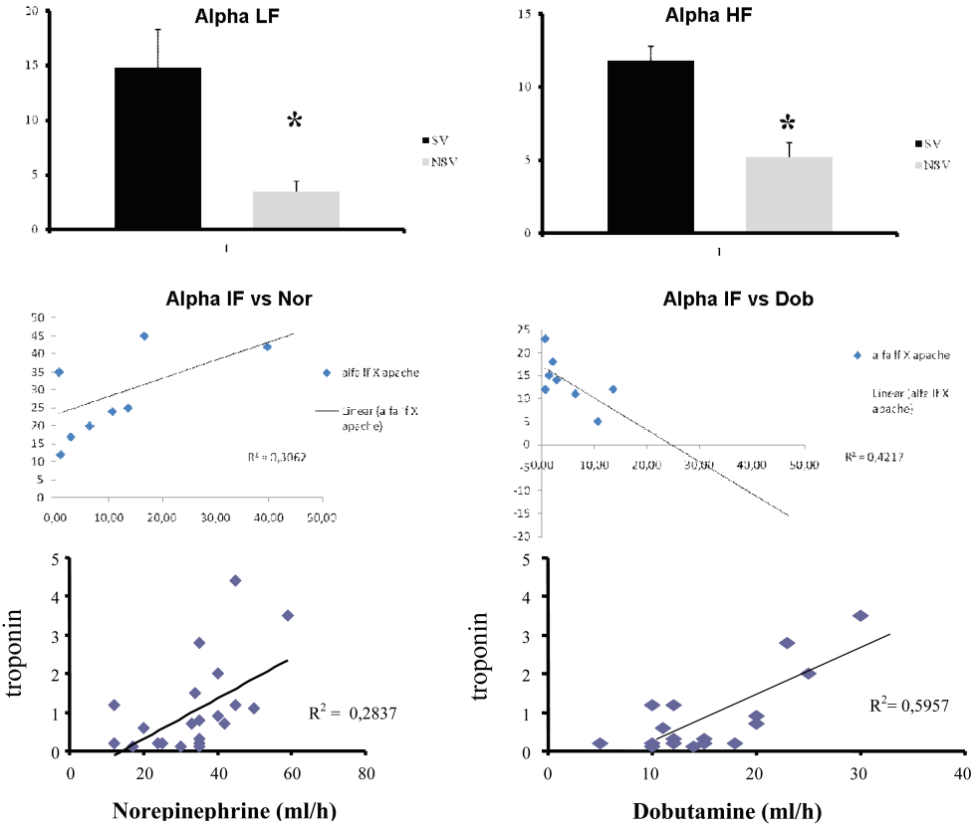


The study included 31 patients, of whom 14 died. Increased troponin levels were related to an increased risk of mortality. The alpha index of HRV low frequency and high frequency indicates that the interaction of baroreceptor in the regulation of heart rate variability with changes in blood pressure had a significant reduction in patients with septic shock and death. The use of dobutamine in patients with septic shock correlates well with troponin levels (*r *= 0.77), while for norepinephine the correlation was poor (*r *= 0.53). The use of dobutamine showed a negative correlation with the low-frequency alpha index (index that assesses the integration of baroreceptor); on the other hand, norepinephine showed a positive correlation. See Table 1 and Figure 1.

**Table 1:** 

Patients, *n*	30
Gender, female	12 (40%)
Age (years)	64 ± 5
Nonsurvivors	14 (47%)
Infectious site	
Lung	18 (60%)
Abdominal tract	5 (15%)
Urinary tract	4 (14%)
Blood	2 (8%)
Nonidentified	1 (4%)
Gram-negative	22 (76%)

## Conclusion

Patients with septic shock have impaired baroreceptor function, and this is correlated with progression to death. Dobutamine is related to higher levels of cardiac damage and higher doses of dobutamine interfere with the responsiveness of the baroreceptor. Moreover, norepinephine has a positive effect on the integration of baroreceptor.

